# Same Performance Changes after Live High-Train Low in Normobaric vs. Hypobaric Hypoxia

**DOI:** 10.3389/fphys.2016.00138

**Published:** 2016-04-19

**Authors:** Jonas J. Saugy, Laurent Schmitt, Anna Hauser, Guillaume Constantin, Roberto Cejuela, Raphael Faiss, Jon P. Wehrlin, Jérémie Rosset, Neil Robinson, Grégoire P. Millet

**Affiliations:** ^1^Faculty of Biology and Medicine, Institute of Sport Sciences, University of LausanneLausanne, Switzerland; ^2^Department of Physiology, Faculty of Biology and Medicine, University of LausanneLausanne, Switzerland; ^3^National School of Mountain Sports/National Ski-Nordic CentrePrémanon, France; ^4^Section for Elite Sport, Swiss Federal Institute of SportMagglingen, Switzerland; ^5^Departmental Section of Physical Education and Sports, University of AlicanteAlicante, Spain; ^6^Swiss Laboratory for Doping Analyses, University of LausanneLausanne, Switzerland

**Keywords:** aerobic exercise, altitude-training camp, crossover study, real altitude, simulated altitude

## Abstract

**Purpose:** We investigated the changes in physiological and performance parameters after a Live High-Train Low (LHTL) altitude camp in normobaric (NH) or hypobaric hypoxia (HH) to reproduce the actual training practices of endurance athletes using a crossover-designed study.

**Methods:** Well-trained triathletes (*n* = 16) were split into two groups and completed two 18-day LTHL camps during which they trained at 1100–1200 m and lived at 2250 m (P_*i*_O_2_ = 111.9 ± 0.6 vs. 111.6 ± 0.6 mmHg) under NH (hypoxic chamber; F_i_O_2_ 18.05 ± 0.03%) or HH (real altitude; barometric pressure 580.2 ± 2.9 mmHg) conditions. The subjects completed the NH and HH camps with a 1-year washout period. Measurements and protocol were identical for both phases of the crossover study. Oxygen saturation (S_*p*_O_2_) was constantly recorded nightly. P_*i*_O_2_ and training loads were matched daily. Blood samples and VO_2*max*_ were measured before (Pre-) and 1 day after (Post-1) LHTL. A 3-km running-test was performed near sea level before and 1, 7, and 21 days after training camps.

**Results:** Total hypoxic exposure was lower for NH than for HH during LHTL (230 vs. 310 h; *P* < 0.001). Nocturnal S_*p*_O_2_ was higher in NH than in HH (92.4 ± 1.2 vs. 91.3 ± 1.0%, *P* < 0.001). VO_2*max*_ increased to the same extent for NH and HH (4.9 ± 5.6 vs. 3.2 ± 5.1%). No difference was found in hematological parameters. The 3-km run time was significantly faster in both conditions 21 days after LHTL (4.5 ± 5.0 vs. 6.2 ± 6.4% for NH and HH), and no difference between conditions was found at any time.

**Conclusion:** Increases in VO_2*max*_ and performance enhancement were similar between NH and HH conditions.

## Introduction

Endurance athletes commonly use altitude training camps with several hypoxic methods to achieve maximal sea-level performance enhancement (Millet et al., 2010). The “Live High—Train Low” (LHTL) method, where athletes live and sleep at altitudes between 2200 and 2500 m and train under 1200 m (Levine and Stray-Gundersen, 1997; Chapman, 2013), is recognized as an effective method that can improves performance in athletes, despite a large inter-subject variability in response (Lundby et al., 2012). More than 15 years of research have revealed that LHTL is an effective training method to enhance sea-level performance in endurance athletes, and it provided 1–3% additional benefit compared with similar normoxic training, although not confirmed by all studies (Siebenmann et al., 2012). These altitude-training camps are conducted under “real” [i.e., hypobaric hypoxia, HH (Stray-Gundersen and Levine, 2008; Chapman et al., 2014; Saugy et al., 2014)] or simulated altitudes [i.e., normobaric hypoxia, NH (Dehnert et al., 2002; Clark et al., 2009; Garvican et al., 2011; Schmitt and Millet, 2012)]. Emerging evidence suggests different physiological responses between these two types of hypoxia (Millet et al., 2012), and it is now admitted that they cannot be used interchangeably (Fulco et al., 2011; Saugy et al., 2014; Coppel et al., 2015; Dipasquale et al., 2015). Short-term exposure in HH seems to induce greater levels of hypoxemia, when compared to NH (Savourey et al., 2003). Likewise, reduced ventilatory responses (Loeppky et al., 1997; Faiss et al., 2013), but higher oxidative stress, combined with impaired nitric oxide bioavailability (Faiss et al., 2013) were reported in HH. Regarding all these differences, pre-acclimatization effectiveness (Fulco et al., 2013) and acute mountain sickness (AMS) scoring (Dipasquale et al., 2015) are logically higher in HH.

Sea-level performance improvement following LHTL may also be different between NH and HH (Bonetti and Hopkins, 2009). Most LHTL studies in HH conditions have reported performance or hematological improvements (Wehrlin et al., 2006; Bonetti and Hopkins, 2009; Chapman et al., 2014; Saugy et al., 2014; Garvican-Lewis et al., 2015), and positive outcomes have been less frequent in NH conditions (Robach et al., 2006b; Bonetti and Hopkins, 2009; Clark et al., 2009; Robertson et al., 2010b), when compared with control (i.e., sea-level) group. However, there is not a sufficient body of knowledge to confirm whether NH or HH induces better performance enhancement after LHTL training camps. It is difficult to compare results from studies with different parameters, such as different hypoxic doses, training loads, temperatures and statistical analyses (Millet et al., 2012; Coppel et al., 2015). There are not any crossover experimental designs to reduce the influence of the confounding factors that influence post-altitude responses and directly compare altitude-induced adaptations and performance changes after LHTL in NH and HH conditions in the same subjects. Athletes and coaches generally consider both hypoxic conditions similar, and it is important to clarify within practical and ecological conditions whether these two types of LHTL training camps may be used interchangeably. Consequently, we designed a crossover study to assess physiological and performance responses in trained athletes during and after LHTL camps matched in the inspired pressure of oxygen (P_*i*_O_2_) in NH or HH conditions. The first phase of the crossover was published previously (Saugy et al., 2014), and results demonstrated better performance enhancement after the altitude training camp conducted under HH conditions. Groups in the present study were crossed to complete the crossover design and reduce an eventual group effect. We hypothesized that the LHTL intervention conducted under HH conditions would produce better performance improvement and greater physiological adaptations than under NH, which is consistent with the results of the first phase of the crossover.

## Methods

### Subjects

Twenty-four well-trained male triathletes participated in the first phase of this study, and 21 male triathletes participated in the second phase. We pooled data across phases to obtain a crossover analysis of 16 subjects who were included in both conditions (*n* = 10 in 2013 and *n* = 6 in 2014 for NH condition, and *n* = 6 in 2013 and *n* = 10 in 2014 for HH condition). The main characteristics of all subjects in the analysis are presented as means ± standard deviation: age 24 ± 4 years, body height 179 ± 5 cm, body weight 70 ± 5 kg, BMI 21.8 ± 1.7 kg.m^2^, VO_2*max*_ 66.3 ± 7.5 mL.kg^−1^.min^−1^, and P_*max*_ 380 ± 48 w. Subjects were included in the NH and HH group in the first phase and switched to the other hypoxia type for the second phase. The washout period between two phases was 1 year. The following inclusion criteria for participation and data analysis were used: (1) a minimum of 5 years of endurance training and frequent participation in endurance competitions; (2) initial ferritin levels >30 μg/l; (3) sufficient training loads during the lead-in period; and (4) participation in both parts of the study (i.e., NH and HH altitude camps). All athletes provided written informed consent to participate in the study. The local ethical committees approved the study (Commission Cantonale Valaisanne d'Ethique Médicale, CCVEM; Agreement 051/09 and French National Conference of Research Ethics Committees; N°CPP EST I: 2014/33; Dijon, France), which corresponded to the two training locations. All experimental procedures conformed to the standards set by the Declaration of Helsinki.

### Study design

**Figure 1:**
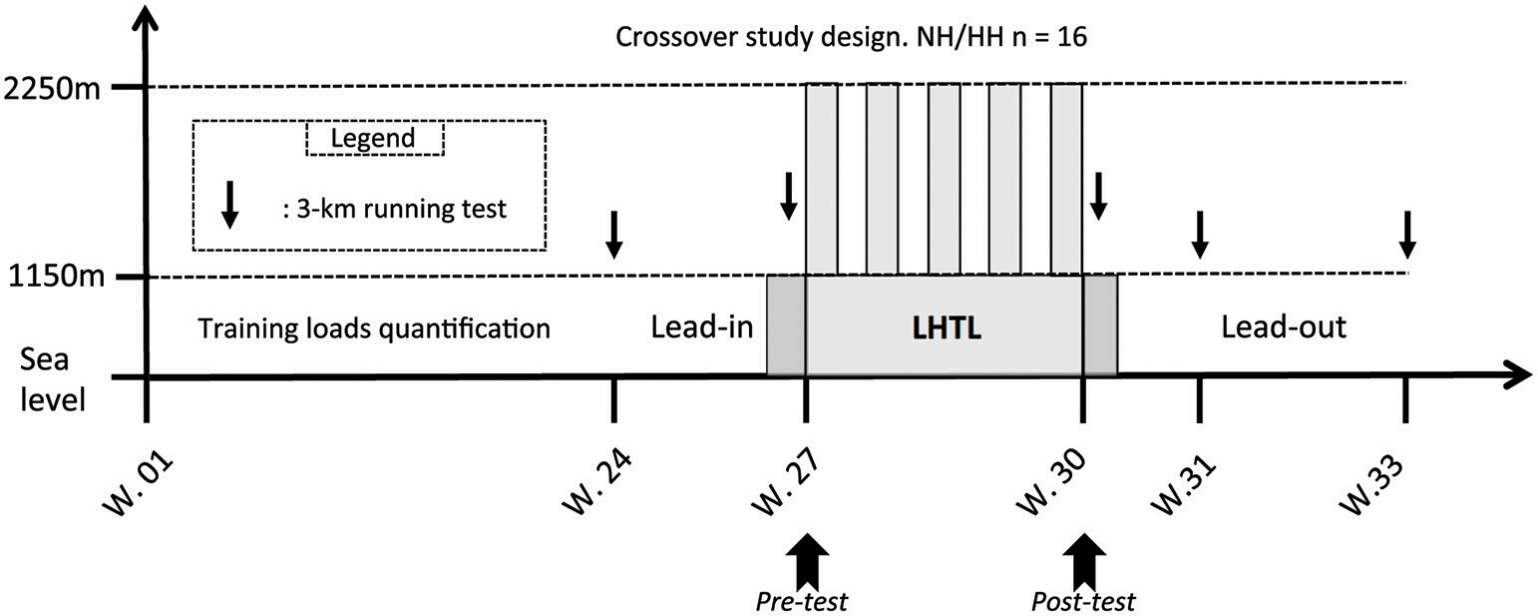
**Overview of the whole protocol conducted in a crossover design in 2 consecutive years**. In horizontal axis the protocol duration of each part in weeks (W) and in vertical axis the testing altitude, including: the 6 months before the lead-in period where the training loads were assessed, the lead-in, the LHTL camp, and the lead-out period. With: 3-km test = the 3-km running tests on the track near sea level made on Pre-, Post-1, Post-7, and Post-21; LHTL = Live High Train Low training camp for normobaric hypoxia (NH) and hypobaric hypoxia (HH). The two dark gray slots before and after the LHTL period correspond to the 2 days of Pre- and Post-tests (i.e., Pre1, Pre2 and Post1, Post2, respectively) at 1150 m.

This study includes the first phase of a crossover design published previously (Saugy et al., 2014). Therefore, values presented here are means of the two phases (i.e., 2013 and 2014) of the crossover study unless specified, and more precise details are provided in the statistics section. Many methodological details are reported elsewhere (Saugy et al., 2014), but these details are also outlined here for the reader's convenience. The experimental design was identical to the first phase of the crossover and consisted of a 33-week period divided into four different phases: (1) 24 weeks of training load quantification at sea level; (2) a 3-week lead-in period also at sea level, where the training loads were quantified, and the training sessions were supervised; (3) an 18-day LHTL training camp under NH or HH conditions; and (4) a 3-week post-altitude period at sea level where the training sessions were also supervised and loads quantified (see Figure 1). The two phases were performed exactly during the same period of the competitive season (July) for the 2 consecutive years, with athletes training in the same club under the supervision of the same coaches. Subjects were assigned to the opposite condition (NH or HH) of the condition they underwent during first phase of the study (i.e., in 2013). They were initially matched based on VO_2*max*_ values that were measured during the Pre-test of the 2013 study. Both groups lived at an altitude of 2250 m under simulated (NH) or real (HH) hypoxic conditions. All subjects trained at an altitude between 1100 and 1200 m. Subjects performed several physiological tests in a well-ventilated laboratory (Prémanon,France, 1150 m) before (Pre-) and immediately after (Post-1) the LHTL camp. Measurements included blood samples, anthropometric measurements, and maximal incremental tests on a cycle ergometer (VO_2*max*_). The Pre- and Post-1 tests were performed in the same order at the same time of day with the same materials for both phases of the crossover study. Subjects performed five 3-km running tests at the following times: prior to lead-in, before LHTL (Pre-), 1 day after LHTL (Post-1), 7 days after LHTL (Post-7), and 21 days after LHTL (Post-21), in exactly the same manner for 2013 and 2014. All 3-km running tests were performed near sea level (between 100 and 390 m).

### Hypoxic exposure

Subjects were exposed to a normobaric hypoxia equivalent to 2250 m during NH conditions, which was obtained by extracting oxygen (i.e., oxygen filtration) from ambient air in hypoxic chambers [inspired oxygen pressure (P_*i*_O_2_) 111.9 ± 0.6 mmHg; inspired oxygen fraction (F_*i*_O_2_) 18.05 ± 0.03%; Barometric pressure (BP) 666.6 ± 3.6 mmHg]. The gas composition in each hypoxic chamber was continuously monitored using oxygen and carbon dioxide analyzers (FIELDBROOK Ltd., London, UK) connected to a central station under the control of an independent and specialized physiologist. The hypoxic chambers were of medium size (15 ± 1 m^2^) and equipped with conventional beds. Two subjects were in each room, and they primarily spent their time sleeping or resting between training sessions. Subjects in NH conditions left chambers 5–6 times daily on average to eat and train. Daily hypoxic dose in NH was 12.7 ± 0.5 h for a total hypoxic exposure of 229.2 ± 5.9 h. The HH group lived in Fiescheralp, Switzerland (2250 m, P_*i*_O_2_ 111.6 ± 0.6 mmHg; F_*i*_O_2_ 20.9 ± 0.0%; BP 580.2 ± 2.9 mmHg) and traveled twice daily to the valley (altitude <1200 m) via cable car for training. The daily hypoxic dose in HH was 17.1 ± 1.7 h for a total hypoxic exposure of 309.9 ± 4.1 h. The hypoxia exposure was monitored daily and recorded manually for both conditions.

### Measurements

#### Training loads

Training consisted of swimming, cycling and running. Two experienced certified coaches supervised and advised athletes during each training session during camps, and intensity and volume were matched for both groups. Training load quantification was performed using “Objective Load Scale” (ECOs; Cejuela Anta and Esteve-Lanao, 2011), which was specially developed for training quantification in triathlons. Briefly, the ECOs were calculated by multiplying the total duration of a training session (in minutes) with a scoring value between 1 and 50, depending on the heart rate-based training zone (1–8) and by a factor of 1.0, 0.75, or 0.5 for running, swimming, or biking, respectively. Daily and weekly training loads (ECOs) of each subject were quantified based on each subject's physical characteristics and training program intensity.

#### Running and maximal oxygen uptake

Running performance was evaluated using 3-km running tests that were completed on a 400-m outdoor synthetic track near sea level. Starts were given individually in a time-trial mode (i.e., 30 s between each subjects) to avoid any group or pacing influences. VO_2*max*_ was tested before and after LHTL (i.e., at Pre-1 or Pre-2 and Post-1, see Figure 1) using an incremental cycling performance test. Subjects were tested on their own bicycles, which were linked to a computerized ergometer system (Cyclus 2®, RBM elektronik automation GmbH, Leipzig, Germany). Workload was increased by 30 W.min^−1^ after a 5-min warm-up period at a workload of 90 W until voluntary exhaustion was reached. Subjects were strongly encouraged to perform until they reached maximal exhaustion. They wore a nose clip and a mouthpiece for breath collection. Oxygen (O_2_) and carbon dioxide (CO_2_) levels were continuously measured and monitored as breath-by-breath values in expired gas (Ultima Cardio 2 gas exchange analysis system, MGC Diagnostics with Breezesuite software, Saint Paul, MN, USA). The flow meter and gas analyzer were calibrated prior to each test. VO_2*max*_ was determined as the highest 30 s average value and based on the standard criteria of maximal exhaustion (VO_2_ plateau, RER >1.1 and incapacity to maintain the exercise load). Maximal power output (P_*max*_) was considered as the load of the last stage completed.

#### Blood samples

Blood samples were taken from the antecubital vein (3 × 4.9 mL EDTA tube®, Sarstedt, Nümbrecht, Germany) either immediately after waking up or before breakfast, twice during each study phase on the first morning during the Pre- and Post-tests (i.e., before and after LHTL, see Figure 1). Blood analyses were conducted using an XT-2000i analyzer® (Sysmex Europe, Norderstedt, Germany) in a Lausanne WADA (World Anti-Doping Agency) accredited laboratory (Lamon et al., 2010). All samples were analyzed in duplicate, and mean values were used for the study. The following hematological parameters were quantified: red blood cells (RBCs), hemoglobin (Hb), hematocrit (Hct), mean cell volume (MCV), mean cell hemoglobin (MCH), mean cell hemoglobin concentration (MCHC), reticulocyte percentage (RET%), and absolute number of reticulocytes (RET#). Regular quality control procedures were applied as required by the standards of WADA-accredited laboratories, and the coefficient of variations (CV) was within the CV limits accepted by the manufacturer for the instrument. Plasma EPO was quantified using an ELISA kit® (Stemcell Technologies, Grenoble, France), and the lower limit of quantification was measured at 1.6 mU/mL. Baseline ferritin was quantified using standard laboratory procedures (Dimension EXL, Siemens Healthcare Diagnostics SA, Zürich, Switzerland) to evaluate subject's iron stores. All athletes were tested for doping by the accredited laboratory according to the biological passport standards to avoid performance enhancement via doping. Determined CVs were always below 15%.

#### Night assessment

S_*p*_O_2_ and HR were recorded nightly from Pre-1 to Post-2 at 0.25 Hz using a wrist oximeter connected to a finger sensor (Wristox 3150® with 8000SM-WO Sensor, Nonin, Plymouth, MN). The oxygen desaturation index (ODI 3%; i.e., the number of times per hour of sleep that the blood's oxygen level drops by 3% or more) has been calculated throughout the periods.

#### Data analysis and statistics

Subjects' data were pooled for each condition from both phases of the study as follows: the NH condition values considered were the pooled values from the NH subjects in 2013 (*n* = 10) and the NH subjects in 2014 (*n* = 6); the same subjects were considered for the HH condition but reversed (*n* = 6 in 2013 and *n* = 10 in 2014; i.e., *n* = 16 for the whole analysis). Data are reported as means and standard deviations of the 16 subjects considered for the crossover analysis. Data were tested for equality of variance (Fisher–Snedecor *F*-test) and normality (Shapiro–Wilk test). When both conditions were met, a two-way ANOVA was performed for repeated measures for each hypoxia condition (NH and HH). To determine the time effects for variables measured on several occasions during camps, pairwise multiple comparison procedures was used (Holm–Sidak method, applied to the S_*p*_O_2_, HR, and training loads). NH and HH were subsequently compared across time (Pre-, Post-1, Post-7, and Post-21) using a two-way ANOVA. When equality of variance or normality was not satisfied (differences in blood parameters tests from Pre- to Post-1 conditions), variables were analyzed for each condition using a Friedman test for repeated measures. To determine time effects, pairwise multiple comparison procedures was used (Bonferroni test). Differences in percentage changes between conditions were tested using a Wilcoxon signed rank sum test (applied to changes in performance and blood parameters). The statistical power of the performed tests concerning the 3000 m performance with alpha = 0.05 was, for the time effect of 1.000, and for the group effect of 0.469. Differences between NH and HH condition at baseline (Pre-) were tested using a Mann-Whitney rank sum test (applied to incremental cycling test parameters and baseline blood sample parameters). Null hypotheses were rejected at *P* < 0.05. All analyses were completed using Sigmaplot 11.0 software (Systat Software, San Jose, CA).

## Results

### Hypoxic doses, P_i_O_2,_ night peripheral oxygen saturation, and heart rate

**Figure 2:**
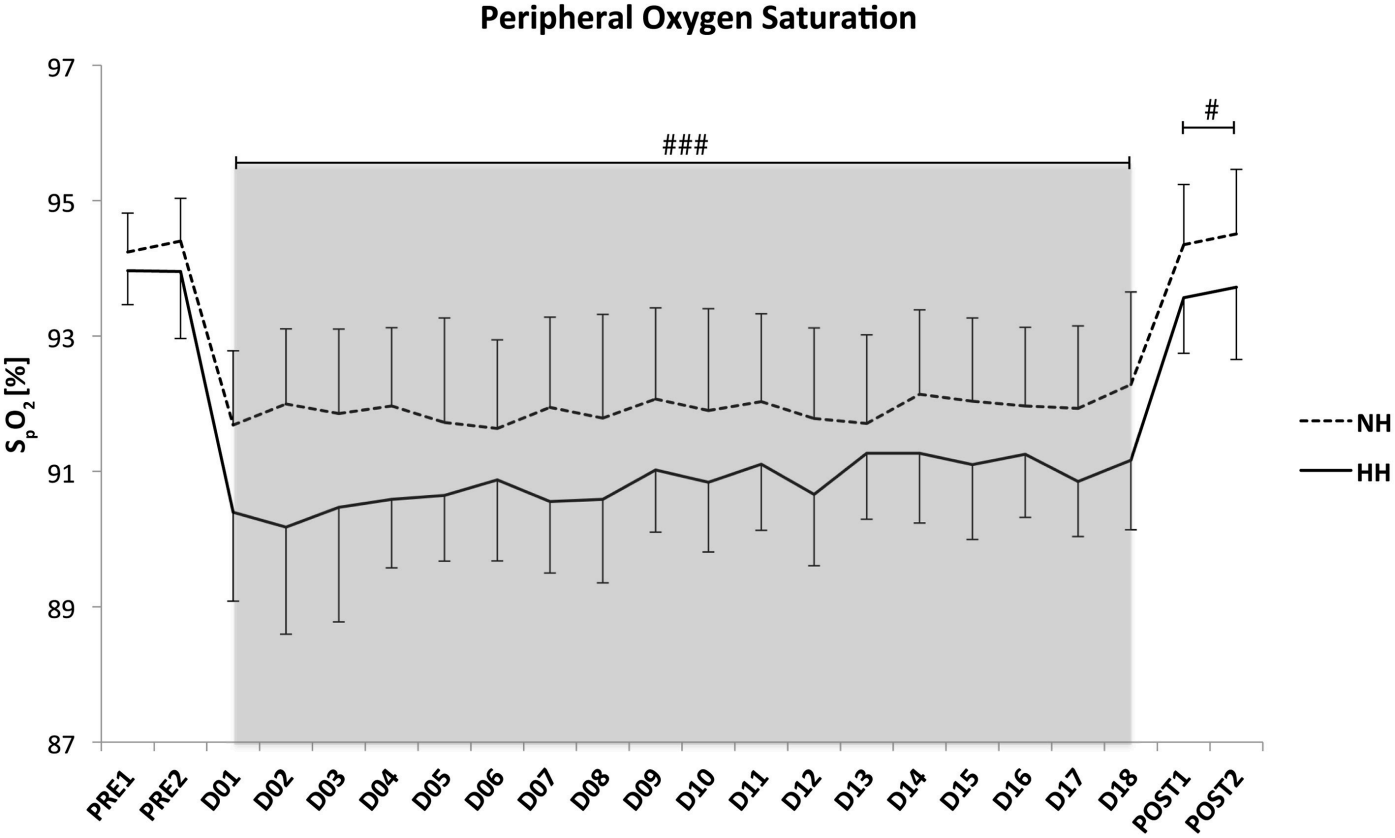
**Mean values of night oxygen pulse saturation (S_***p***_O_**2**_) for the crossover data**. Data are presented in mean ± standard error. Pre1-Pre2: measurements before the camps (1150 m, Prémanon, France); D01–D18: measurement during the camps (NH: hypoxic room in Prémanon, France; HH: Fiescheralp, Switzerland); Post1-Post2: measurements after the camps (1150 m, Prémanon, France). #*P* < 0.05, ###*P* < 0.001 for differences between conditions.

Daily hypoxic dose (12.7 ± 0.5 vs. 17.1 ± 1.7 h, *P* < 0.001) and total hypoxic exposure (229.2 ± 5.9 vs. 309.9 ± 4.1 h, *P* < 0.001) were lower in NH than in HH. The average P_*i*_O_2_ values were not different between conditions (111.9 ± 0.6 vs. 111.6 ± 0.6 mmHg, for NH and HH). The nightly average of HR was higher for NH than for HH (51 ± 1 vs. 48 ± 2 bpm for NH and HH, *P* < 0.001), and these values stayed higher when returning to 1200 m in Prémanon during the two nights of post-test (51 ± 2 vs. 46 ± 2 bpm, for NH and HH, *P* < 0.001). Nightly S_*p*_O_2_ values were similar between two groups during the control nights (i.e., the two nights at 1150 m before LHTL camps, Pre-1 and Pre-2), but values were higher in NH than in HH during the entire camp (D1–D18; 92.4 ± 1.2 vs. 91.3 ± 1.0%, for NH and HH, *P* < 0.001). These values remained higher (*P* < 0.05) during the two nights at Post-1 (94.4 ± 0.9 vs. 93.6 ± 0.9%, for NH and HH, *P* < 0.05). All values are presented in Figure 2. In addition, the ODI 3% was significantly lower for NH than HH throughout the hypoxic nights (9.9 ± 1.6 vs. 15.1 ± 3.5, *P* < 0.001).

### Training loads

No difference was found in daily training loads during the lead-in (3 weeks prior LHTL camps; 79.8 ± 22.7 vs. 87.4 ± 23.1 ECOs) or the lead-out (3 weeks following the LHTL camps; 164.4 ± 20.2 vs. 173.9 ± 24.1 ECOs; see Figure 1) periods between NH and HH, respectively. No difference was found in daily training loads (226.7 ± 56.5 vs. 214.5 ± 56.4 ECOs for NH and HH groups) between the two groups during the 18-days LHTL camps in both conditions of the crossover. Finally, weekly training loads monitored during the 24 weeks prior to the study were not different between groups (979 ± 207 vs. 1135 ± 98 ECOs for NH and HH group, respectively). Daily training loads during the LHTL in phase 2 were reduced compared to phase 1 (232.2 ± 27.2 vs. 220.3 ± 31.4 for NH and 217.3 ± 48.1 vs. 211.4 ± 21.0 ECOs for HH, *P* < 0.05).

### 3-km performance test

**Figure 3:**
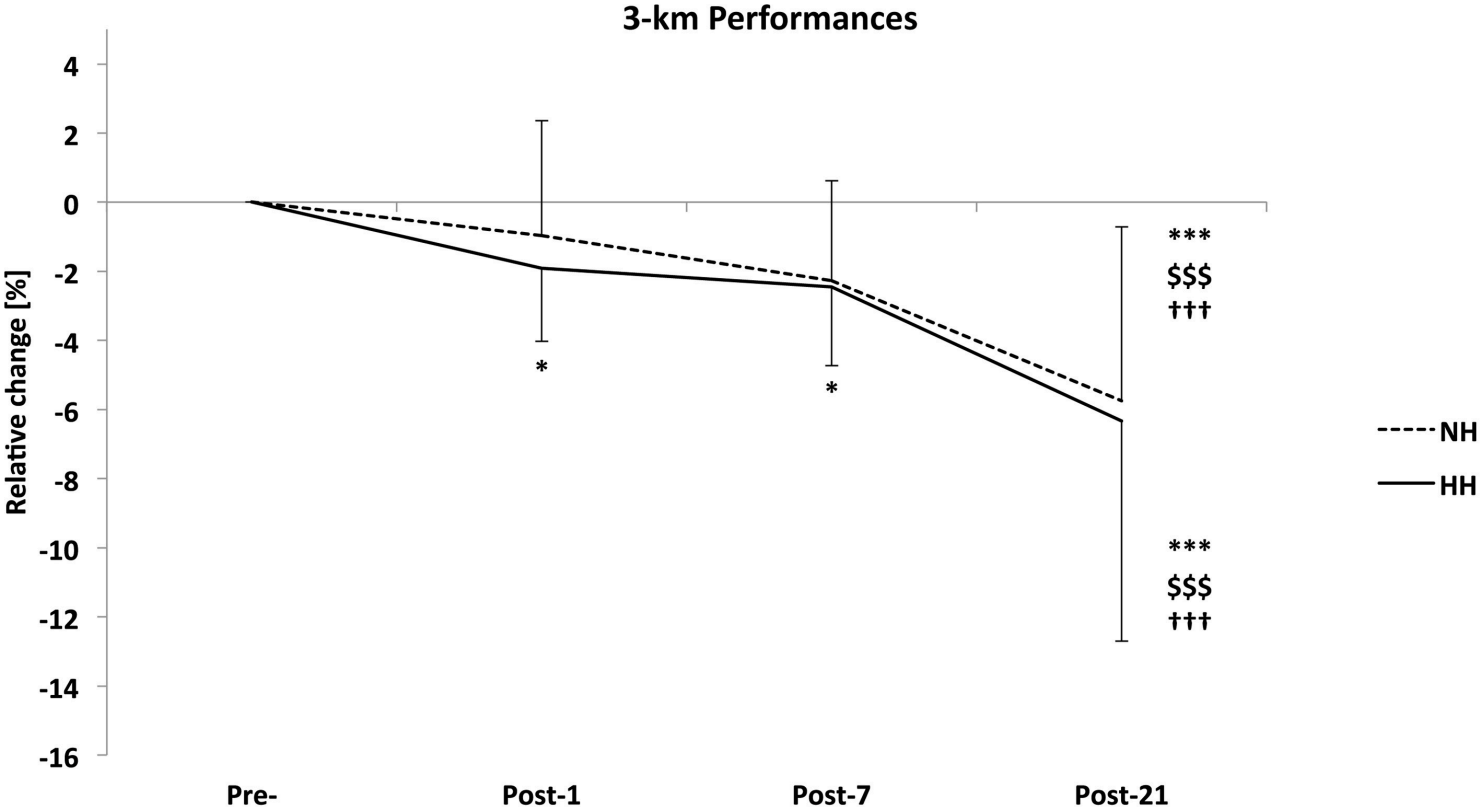
**Relative change in 3-km run time from Pre- to Post-1, Post-7, and Post-21 as determined on a running track near sea level for the normobaric hypoxia (NH) and hypobaric hypoxia (HH) conditions (in %) for the crossover (***n*** = 16)**. Data are mean ± standard error ^*^P < 0.05 and ^***^P < 0.001 for differences with Pre-; ^$$$^*P* < 0.001 for differences with Post-1; ^†††^*P* < 0.001 for differences with Post-7.

The 3-km performance was significantly increased to a larger extent in the HH group than in the NH group at Post-21 in the first phase of the study (−1.2 ± 2.9 vs. −3.2 ± 3.8%, for NH and HH, *P* < 0.05). Performance in the second phase (i.e., 2014) increased from Pre- to Post-1 (−3.3 ± 2.0 vs. −3.9 ± 2.9%, for NH and HH, *P* < 0.01), Post-7 (−2.7 ± 3.1 vs. −2.6 ± 3.6%, for NH and HH, *P* < 0.05), and Post-21 (−8.4 ± 4.1 vs. −9.1 ± 6.1%, for NH and HH, *P* < 0.01). Performance increased from Post-1 and Post-7 to Post-21 for both conditions. However, no difference was noted between NH and HH groups at any time. The crossover demonstrated that performance increased from Pre- to Post-1 (**–**1.92%, *P* < 0.05) and Post-7 (**–**2.44%, *P* < 0.05) for HH but not in NH (–0.97 and -2.27% from Pre- to Post-1 and Post-7, respectively, ns). And it increased from Pre- to Post-21 (*P* < 0.001), Post-1 to Post-21 (*P* < 0.001), and Post-7 to Post-21 (*P* < 0.001) for both conditions. However, no difference was noted between conditions at any time (Figure 3). We found important inter-individual differences between both conditions, i.e., during 2 successive years. For example subject n°1 decreased his performance time at Post-21 by -3.6% in NH vs. -7.4% in HH. Subject n°6 increased his performance time at Post-21 by 1.9% in NH and decreased it by -12.1% in HH.

### Maximal test on cycle ergometer

Subjects increased their power output and maximal oxygen uptake values immediately after altitude training camps in both NH and HH, but without any difference between conditions. Table 1 presents all values. Ventilation and heart rate at the submaximal work rate of 190 ± 24 W corresponding to 50% PPO pre- values have been measured. HR decreased to the same extent from Pre- to Post-1 (-6 ± 3 vs. −6 ± 2 bpm) and ventilation did not change significantly (2.5 ± 2.2 vs. −1.7±7.9 L.min^−1^) in NH vs. HH, respectively.

**Table 1:** **Main parameters measured during the incremental test on ergocycle for normobaric hypoxia (NH) and hypobaric hypoxia (HH) group before (Pre-) and after (Post-) the camps**.

		**Pre-**	**Post-**	**Delta %**
VO_2_*max* [ml.O_2_.kg.min^−1^]	NH	65.2±6.3	68.3±5.0^***^	4.9±5.6
	HH	66.7±8.5	68.8±2.4^**^	3.2±5.1
HR_*max*_ [bpm.min^−1^]	NH	191±8	191±6	0.4±2.2
	HH	190±7	189±6	−0.5±1.7
P_*max*_ [W]	NH	362±45	385±33^***^	7.1±6.6
	HH	397±46	411±39^***^	4.9±5.1
VE_*max*_ [l.min^−1^]	NH	180.1±17.4	185.4±15.1	3.6±10.5
	HH	192.3±31.7	195.2±21.7	2.3±7.7

Physiological parameters during the incremental test on ergocycle; VO_2max_, maximal oxygen uptake; HR_max_, maximal heart rate; P_max,_ maximal power output; VE_max,_ maximal ventilation. Data are mean ± SD;

** P < 0.01 and

*** *P < 0.001 for differences between Pre- and Post-*.

### Hematological parameters

EPO values decreased significantly to the same extent from Pre- to Post-1 in both conditions (-30 ± 25 and -36 ± 21% for NH and HH, *P* < 0.001). No significant changes were observed in the other parameters. Table 2 presents all values.

**Table 2:** **Main parameters measured with blood analyses for normobaric hypoxia (NH) and hypobaric hypoxia (HH) group before (Pre-) and after (Post-) the camps**.

		**Pre-**	**Post-**	**Delta %**
EPO [mU/mL]	NH	5.12±2.57	3.08±1.55^***^	−33.6±27.3
	HH	4.48±1.45	3.02±0.71^***^	−35.7±19.7
RBC [u/μl]	NH	5.21±0.49	5.29±0.46	1.7±7.3
	HH	5.24±0.44	5.29±0.38	1.1±4.6
Hb [g/dl]	NH	15.38±1.23	15.99±1.05	4.3±6.7
	HH	15.44±1.08	15.75±1.01	2.4±5.1
Hct [%]	NH	45.52±3.31	46.70±3.10	2.9±7.1
	HH	45.51±3.16	46.44±2.59	2.5±3.7
MCV [fl]	NH	87.56±4.10	88.53±3.48	1.15±1.3
	HH	87.19±3.46	87.96±3.30	0.9±1.4
MCH [pg]	NH	29.56±1.02	30.30±1.22	2.5±1.8
	HH	29.50±1.12	29.84±1.26	1.2±1.8
MCHC [g/dl]	NH	33.78±0.81	34.25±0.92	1.4±1.7
	HH	33.83±0.85	33.92±0.91	0.3±2.1
RET [%]	NH	1.14±0.53	1.13±0.27	6.3±31.1
	HH	1.09±0.28	1.09±0.36	3.8±31.8

Blood and hemoglobin parameters; EPO, erythropoietin; RBC, red blood cells; Hb, hemoglobin; Htc, hematocrit; MCV, mean cell volume; MCH, mean cell hemoglobin; MCHC, mean cell hemoglobin concentration; RET, reticulocytes. Data are mean ± SD;

*** *P < 0.001 for differences between Pre- and Post-*.

## Discussion

This study is the first crossover study to compare physiological and performance responses during and after an 18-days LHTL altitude camp conducted in NH or HH conditions. The crossover design incontestably increased the statistical power of the present analysis compared with the first phase of this study (Saugy et al., 2014). Lower peripheral oxygen saturation levels during the night and longer hypoxic exposure were noted in HH than in NH. However, no differences in blood parameters, maximal power output or VO_2max_ were observed between hypoxic conditions. Both conditions directly induced performance enhancement 7 or 21 days after the camps. However, no differences were observed between NH and HH in sea level performances.

The second phase (i.e., 2014) and crossover 3-km running performances were not different between conditions at any measurement point (Figure 3), which is unlike the first phase of the study. Both conditions induced performance enhancements Post-1, Post-7, and Post-21 in 2014 and at Post-21 when considering the crossover. VO_2*max*_ and performance increased to the same extent for NH and HH conditions. So the VO_2*max*_ increase itself could explain at least partly the enhanced performance. Moreover, there is currently a large debate about running economy and efficiency when returning from altitude camps (Schmitt et al., 2006; Lundby et al., 2007; Chapman, 2013). In the present study VO_2_ and ventilation were unchanged while HR decreased to the same extent during a submaximal cycling test in both groups. These later results show that cycling efficiency was not modified. It is therefore likely that running economy did not change significantly and did not influence the running performance enhancement. This evolution is consistent with the established insight for the development of performance after altitude training camps described by Sinex et al. (Sinex and Chapman, 2015) and drawn from Millet et al. (2010). These studies have demonstrated initial improvements in performance (days 1–7) and a higher plateau in performance (days 18–20 or more). The result that improvements were significant for only HH at Post-1 and Post-7 in the crossover was clearly influenced by NH data of 2013. A group effect was observed in the first phase, and NH subjects in 2013 did not assimilate the combination of training load and hypoxic dose as well as the other group. The training content and load were strictly similar between the two groups during each phase but were adjusted from phase 1 to phase 2 by coaches (probably from their experience of phase 1). The main change consisted in a reduced training load in phase 2 (232.2 ± 27.2 vs. 220.3 ± 31.4 for NH and 217.3 ± 48.1 vs. 211.4 ± 21.0 ECOs for HH, in phase 1 vs. phase 2) and a different periodization. Of interest is that a similar training adjustment has been performed between successive studies conducted in a chronological order at the same location; i.e., with elite Nordic skiers (Robach et al., 2006a), swimmers (Robach et al., 2006b), and distance runners (Brugniaux et al., 2006). The crossover design tone down this tendency, but it highlights the considerable importance of inter-individual variations in responses to altitude training (Friedmann et al., 2005; Garvican et al., 2010; Chapman, 2013; Sinex and Chapman, 2015). Of interest is the observed intra-individual variability between successive years, in line with a previous case study (Garvican et al., 2007). This inter- or intra-subjects variability between the two phases raises questions about the physiological basis of highly variables findings from previous published LHTL studies (Pialoux et al., 2009; Robertson et al., 2010a; Nordsborg et al., 2012; Robach et al., 2012; Siebenmann et al., 2012; Garvican-Lewis et al., 2013). With small effects and sample sizes, added to the large among of confounding factors (i.e., training loads, subjects training level, food supplies, sleep…), the probability of type 2 errors has been often under-considered.

The daily exposures are consistent with previous studies in normobaric or hypobaric hypoxia with 8–12 h.d^−1^ (Roberts et al., 2003; Saunders et al., 2004) and 18 h.d^−1^ (Levine and Stray-Gundersen, 1997; Wehrlin and Marti, 2006) in NH and HH, respectively. Night peripheral oxygen saturation was lower for HH than for NH from the beginning to the end of the camps, and it remained lower after returning to Prémanon (1150 m) for the Post-tests (Figure 2). This result confirmed the results of the first phase of the crossover, and it is consistent with previous studies (Savourey et al., 2003, 2007; Self et al., 2011) using short exposures and higher altitudes (<1 h, 4500–7620 m). No difference was reported in longer exposure (up to 24 h, from 3000 to 4564 m) studies (Roach et al., 1996; Loeppky et al., 2005; Miyagawa et al., 2011; Faiss et al., 2013). However, the hypoxic exposure was always shorter than in the present study. To our knowledge, no study has directly compared NH and HH conditions in LHTL camps using a crossover design. Potential mechanisms underlying this difference in the nocturnal S_*p*_O_2_ found in the present study were reported previously (Saugy et al., 2014). Briefly, a stronger pulmonary vasoconstriction in HH, which was induced by the modified fluid circulation and trans-alveoli-capillary membrane flux under the influence of barometric pressure, may lead to decreased pressure gradient and oxygen diffusion (Levine et al., 1988; Loeppky et al., 2005; Millet et al., 2012). In addition, the ODI 3%, a reliable indicator of apnea/hypopneas index was calculated throughout all nights and indicated larger sleep disordered breathing in HH than NH. This is in line with previous results from Heinzer et al. (2013) who has reported more hypopneas with polysomnography analyses in HH compared to NH. However, the present difference in ODI 3% seems not clinically relevant (much lower than reported values in clinical groups) for inducing difference in performance enhancement between conditions. Moreover, Goodall et al. (2014) recently found that the integrity of the corticospinal system is modified after 2 weeks at 5260 m, which potentially reduces fatigue level observed in acute hypoxia and might be a contributor to increased performance following acclimatization. Moreover, adaptive changes have been observed after 3 h of NH exposure (Rupp et al., 2012). Thus, it seems that a time-dependent effect on the central nervous system exists. These central nervous system adaptations are likely influencing the observed changes in performance. However, these mechanisms have not been investigated in the present study conducted under lower altitudes.

Differences in daily and total hypoxic exposures were found between conditions (13 vs. 17 h.d^−1^ and 230 vs. 310 h for NH and HH), but our aim was to compare the two LHTL camps in “real conditions” in ecological ways. Moreover, this hypoxic doses difference is not the main factor for the S_*p*_O_2_ difference between NH and HH since S_*p*_O_2_ was lower for HH from the first night of exposure and remained stable throughout the camps (see Figure 2).

Hematological parameters evolved to the same extent in both conditions, which is consistent with recent studies using natural (Garvican et al., 2012; Garvican-Lewis et al., 2013, 2015; Saugy et al., 2014) or simulated altitudes (Wehrlin et al., 2006; Gore et al., 2013; Saugy et al., 2014) in LHTL protocols, but none of these studies have directly compared NH and HH. Interestingly, most of the studies on “altitude acclimatization” (e.g., conducted with untrained lowlanders) failed to demonstrate altered erythrocyte volume for up to 3 weeks below 4000 m (Sawka et al., 2000). These authors reported that “physical activity modulates the erythrocyte volume expansion during altitude acclimatization” and that elite athletes engaged in aerobic training, despite a large inter-individual variability, might have larger benefits from the same hypoxic dose, due to genetically inherited factors that may modulate the hypoxic ventilatory drive, Hb P50, or erythropoietin responsiveness to hypoxia. Most of these points remain unresolved and were beyond the scope of the present study. Nevertheless, the LHTL camps in NH or HH did not affect hematological parameters, except serum EPO concentrations (Table 2). However, the higher the Hbmass value at the start of the hypoxic exposure, the lower the Hbmass increase (Robach and Lundby, 2012). This result is consistent with previous studies from Dehnert et al. (2002) and Robach et al. (2006b), who have observed no changes in primary hematological parameters. EPO concentrations significantly fell when the subjects returned to 1150 m for the Post-tests, but this drop was not different between conditions in the crossover, unlike the first phase of the study. A drop in EPO after return to normoxia following continuous hypoxic exposure was reported previously (Milledge and Cotes, 1985; Savourey et al., 1996, 2004; Risso et al., 2007; MacNutt et al., 2013). The lower serum EPO concentration found after the camp in the present study may be explained by the oscillating nature of LHTL, which was suggested by Garvican et al. (2012), but the underlying mechanisms are not clear. However, MacNutt et al. (2013) has provided indirect evidence of neocytoloysis and an assumption of the mechanisms in a study with mice: a decrease in EPO mRNA within 1 h of hypoxia cessation combined with neocytolysis, whereby the most recently formed erythrocytes are targeted for destruction and phagocytized by macrophages in the spleen.

## Strength and limitations

The use of a crossover design increases statistical power, and it is of great importance because of the large inter-subject variations due to hypoxia (Coppel et al., 2015). It is even more important when comparing normobaric and hypobaric hypoxia because of the slight nature of their physiological differences. However, considering the statistical power (i.e., 0.469) for the group effect on the 3000 m performances, we cannot exclude the presence of type-2 error. Thus, there is still a possibility of performance difference between NH and HH, despite the crossover-designed protocol. This study is the first crossover study to compare prolonged altitude training in NH and HH. The present study compared the physiological and performance differences between NH and HH during and after a 3-week LHTL conducted under “real” conditions (i.e., daily exposures based on the literature and real training sessions supervised by coaches). The aim of this study was not to test the effectiveness of LHTL training alternatives. It is likely that the large performance enhancement following the LHTL period was due to the intensified training during both LHTL and lead-out periods. The high training loads during the LHTL camp could be the main stimulus leading to performance gain during the lead-out period. Given the increase in training loads and the lack of a control group, we cannot evaluate whether hypoxic exposure/acclimatization actually contributed to the performance enhancement. Moreover, considering the fact that there was no control group, we cannot rule out that there is a strong placebo effect that would influence partly the performance enhancement in the athletes. It is a serious limitation in the present study since we cannot appreciate the magnitude of this placebo effect and if it was different between NH and HH conditions. In addition, considering the statistical power above-mentioned, further studies are needed with larger sample sizes to completely answer the research question.

Athletes were well trained, and training loads were not different between conditions. Training loads were quantified 6 months prior the study and supervised during the whole protocols for both phases of the crossover. The “real life” parameters of this study induced significant hypoxic dose differences between conditions. One cannot exclude that the slight physiological differences found between NH and HH would also appear with same hypoxic doses. On the other hand, we have to consider that simulated vs. real altitude might have produced different results if we had compared them at equal hypoxemia doses. Since the condition × time interaction was not significant, one cannot report from a statistical point of view that a condition was more efficient than the other one. However, from a practical and coaching point of view, the 0.95, 0.17, and 0.58% larger performance improvement in HH compared to NH (from Pre- to Post-1, Post-7, and Post-21, respectively) are not negligible. Nevertheless, the aim of the present study was not a true comparison between these two hypoxic conditions, which would have requires equal stimulus levels. This crossover study confirmed that NH and HH involve different physiological adaptations but elicit similar performance improvements when using LHTLs of the same duration. The hypoxic dose and/or the altitude level should be adjusted to individual athlete responses, e.g., the night S_*p*_O_2_, to achieve the highest performance improvements. Obviously, the NH condition is more convenient for this purpose. Further investigations should focus on the individualization of the training and hypoxic exposure. Nevertheless, it is important to take into consideration that the group effect from the larger NH cohort in 2013 could be driving the results. Since the crossover is not perfectly balanced (with 10 and 6 instead of 8 and 8) the crossover design could not “cancel” this tendency.

## Conclusion

The present crossover study provided a further step to compare normobaric and hypobaric hypoxia. The results confirmed that NH and HH are definitely not interchangeable when several physiological responses are considered, as suggested previously (Fulco et al., 2011; Millet et al., 2012; Saugy et al., 2014; Coppel et al., 2015). Despite differences in the stimulus that occur when using these two different methods in real life, we report no observable differences in responses to NH vs. HH.

However, the hematological responses and performance improvements post-LHTL were similar. Each hypoxic condition has advantages and drawbacks from a practical point of view. The HH condition leads to longer hypoxic doses for a given training period (i.e., 18 days in this study), which may fit easier into a complex training plan with elite athletes, but the logistical constraints, and the cost may be detrimental. In contrast, NH condition allows athletes and trainers to individualize the hypoxic stimulus. It may be interesting to adjust the hypoxic dose by modifying the time spent in the room or the altitude setting to athletes' physiological responses and training level. However, training camps conducted under normobaric hypoxia require longer periods of time to achieve sufficient hypoxic doses because of the lower amount of time spent in hypoxia than under HH.

## Author contributions

JS, LS, and GM made substantial contributions to the conception and design of the work, the acquisition, analysis, and interpretation of data for the work; they drafted the work and revising it critically for important intellectual content. AH, GC, RC, RF, JW, JR, NR made the acquisition, analysis, or interpretation of data for the work and revising it critically for important intellectual content. JS, LS, AH, GC, RC, RF, JW, JR, NR, and GM gave their final approval of the version to be published and agreement to be accountable for all aspects of the work in ensuring that questions related to the accuracy or integrity of any part of the work are appropriately investigated and resolved.

### Conflict of interest statement

The authors declare that the research was conducted in the absence of any commercial or financial relationships that could be construed as a potential conflict of interest.
